# Convergent Evolution of Slick Coat in Cattle through Truncation Mutations in the Prolactin Receptor

**DOI:** 10.3389/fgene.2018.00057

**Published:** 2018-02-23

**Authors:** Laercio R. Porto-Neto, Derek M. Bickhart, Antonio J. Landaeta-Hernandez, Yuri T. Utsunomiya, Melvin Pagan, Esbal Jimenez, Peter J. Hansen, Serdal Dikmen, Steven G. Schroeder, Eui-Soo Kim, Jiajie Sun, Edward Crespo, Norman Amati, John B. Cole, Daniel J. Null, Jose F. Garcia, Antonio Reverter, William Barendse, Tad S. Sonstegard

**Affiliations:** ^1^CSIRO Agriculture and Food, Saint Lucia, QLD, Australia; ^2^US Dairy Forage Research Center, United States Department of Agriculture, Agricultural Research Service, Madison, WI, United States; ^3^Unidad de Investigaciones Zootécnicas, Facultad de Ciencias Veterinarias, Universidad del Zulia, Maracaibo, Venezuela; ^4^Department of Preventive Veterinary Medicine and Animal Reproduction, School of Agricultural and Veterinarian Sciences, São Paulo State University, São Paulo, Brazil; ^5^International Atomic Energy Agency, Collaborating Centre on Animal Genomics and Bioinformatics, Araçatuba, Brazil; ^6^Department of Animal Science, University of Puerto Rico-Mayagüez, Mayagüez, Puerto Rico; ^7^Department of Animal Sciences, University of Florida, Gainesville, FL, United States; ^8^Department of Animal Science, Faculty of Veterinary Medicine, Uludağ University, Bursa, Turkey; ^9^Animal Genomics and Improvement Laboratory, United States Department of Agriculture, Agricultural Research Service, Beltsville, MD, United States; ^10^Recombinetics, Inc., Saint Paul, MN, United States; ^11^College of Animal Science, South China Agricultural University, Guangzhou, China; ^12^Department of Support, Production and Animal Health, School of Veterinary Medicine, São Paulo State University, São Paulo, Brazil

**Keywords:** cattle, evolution, convergent, livestock, SNP, NGS, prolactin receptor

## Abstract

Evolutionary adaptations are occasionally convergent solutions to the same problem. A mutation contributing to a heat tolerance adaptation in Senepol cattle, a New World breed of mostly European descent, results in the distinct phenotype known as slick, where an animal has shorter hair and lower follicle density across its coat than wild type animals. The causal variant, located in the 11^th^ exon of *prolactin receptor*, produces a frameshift that results in a truncated protein. However, this mutation does not explain all cases of slick coats found in criollo breeds. Here, we obtained genome sequences from slick cattle of a geographically distinct criollo breed, namely Limonero, whose ancestors were originally brought to the Americas by the Spanish. These data were used to identify new causal alleles in the 11^th^ exon of the *prolactin receptor*, two of which also encode shortened proteins that remove a highly conserved tyrosine residue. These new mutations explained almost 90% of investigated cases of animals that had slick coats, but which also did not carry the Senepol slick allele. These results demonstrate convergent evolution at the molecular level in a trait important to the adaptation of an animal to its environment.

## Introduction

Convergent evolution occurs when species adapt to a given condition through the same or very similar phenotype using different pathways, genes, or mutations. Convergence might occur in broad evolutionary sweeps, such as the evolution of wings in different phyla, or through the manifestation of similar morphologies, such as found in burrowing animals of the same class (e.g., aardvarks and anteaters), or via different mutations in the same gene or gene pathway to alter skin or feather colors across a range of animals. It is not always possible to say whether an example of convergence is due to a single gene or due to changes at several genes. For instance, although the color mutations involving melanin generally involve mutations in a small set of genes (Yang et al., 2016), body shape differences are frequently correlated to changes in one or more genes within sets of 100s of genes.

The thermal environment is an important source of selective pressure, and has resulted in the generation of many different humidity- and temperature-related adaptations in Eutherian mammals. Adaptations-related to extremely cold environments include thick layers of subcutaneous fat, blubber, and long hair or fur for insulation. Heat-adapted animals, by contrast, tend to have short coats, fat restricted to specific parts of the body, and efficient mechanisms to shed heat. Cattle are no exception to this, with temperate, taurine cattle (*Bos taurus*) exhibiting, among other traits, long hair with a wooly undercoat, thicker subcutaneous fat layers, and larger digestive tracts in which heat generating rumination can occur. This is contrasted by the traits exhibited by the tropically adapted, indicine cattle (*Bos indicus*), which have short hair coats, large skin folds, increased heat flow from the body to the skin and reduced metabolic rate (Turner and Schleger, 1960). Crosses of indicine with taurine cattle show a wide range of intermediate types suggesting that these traits have a multifactorial genetic basis. One result is hybrid cattle produced by successive generations of crossbreeding and selection that have smooth or slick coats due to their indicine ancestry.

Nearly 500 years ago a cattle was first introduced in the Americas from the Iberian Peninsula (Martinez et al., 2012). These cattle that went to create the criollo breeds were moved from a mainly temperate or Mediterranean environment into the tropics, much warmer and humid than they were used to. Now, after years of local adaptation, there currently exist an extensive collection of criollo breeds of cattle that are locally adapted to their environment, some to extreme harsh conditions (Martinez et al., 2012; Anderson et al., 2015). The high diversity among the criollo cattle poses an opportunity to better understand the biological forces driving these local adaptations and open the possibility to make use of it in a commercial enterprise either for beef or milk production.

Recently, a single mutation in *prolactin* (*PRL*) and another in *prolactin receptor* (*PRLR*) was shown to have a major genetic effect on hair length and coat structure characteristics of cattle (Littlejohn et al., 2014). In particular, the very smooth coats of the Senepol breed (henceforth termed the “slick” phenotype), a composite of taurine breeds developed in Saint Croix in the United States Virgin Islands (Flori et al., 2012), was shown to result from a frameshift deletion within the last exon of *PRLR* that truncates a portion of the cytoplasmic domain of the protein (Littlejohn et al., 2014). Slick coat can be considered an indicative or indirect phenotype to some highly important production traits, as it is consistently associated to higher thermotolerance and higher milk production on cross-breeds under tropical environment (Dikmen et al., 2014; Casas and Kehrli, 2016; Carabano et al., 2017). It was not known, however, if the mutation described by Littlejohn et al. (2014) on Senepol and its crosses was the only mutation leading to slick coats.

Among the diverse range of criollo cattle there are some that carry slick coats, but do not always carry the previously identified *PRLR* mutation. We postulated that other genetic variants were causing the slick phenotype in these non-concordant cattle. Our search for causal mutations producing the slick phenotype in criollo breeds revealed a surprising degree of mutational plasticity within the cytoplasmic domain of the encoded protein of *PRLR*. In this study, we describe these results and show evidence of convergent evolution for smooth coats in European-derived cattle that have been bred in the tropics.

## Materials and Methods

### Animals Samples and Phenotypes

Coat characteristics of animals with slick and non-slick phenotypes were scored using previously published methods (Turner and Schleger, 1960; Mariasegaram et al., 2007; Landaeta-Hernández et al., 2011; Huson et al., 2014; Porto-Neto et al., 2014). DNA was obtained from a variety of sources including blood, hair, and semen, and extracted using previously published methods (Harrison et al., 2012; Huson et al., 2014; Porto-Neto et al., 2014). Collection and analysis of the Australian samples have been reported previously (Harrison et al., 2012; Porto-Neto et al., 2014).

The SNP discovery followed the genome-wide association analyses being performed using Limonero cattle. The called validation sample (*n* > 800) included cattle from several parts of the world to which coat phenotypes had been recorded, these included Limonero and Carora (Venezuela), Tropical Composite and Senepol crosses (Australia), Holstein crosses (Puerto Rico and United States – Florida).

### Genome-Wide Association on Limonero Cattle

The genome-wide association analyses were run using 20 slick and 53 non-slick Limonero animals, and 519,004 autosomal SNP that passed quality control [Hardy–Weinberg equilibrium (*p* > 10^-5^), minimum call rate of 95% and a minor allele frequency (MAF) of at least 5%]. Markers were tested for association using the regression model implemented in the –mlma option of GCTA v1.21 (Yang et al., 2011). Briefly, a liability-threshold model was used, where the observed slick status was assumed to result from a dichotomization of a standard normal latent variable. Phenotypes on the observed 0-1 risk scale were regressed onto genotypes assuming **y** ∼ MVN(**1**μ + **z**_i_α_i_, **G**σ_u_^2^ + **I**σ_e_^2^), where **y** is the vector of phenotypes, **1** is a vector of 1 s, μ is the overall mean, **z**_i_ is a centered genotype vector at tested marker *i*, α_i_ is the allele substitution effect of tested marker *i*, **G** is the realized genomic relationship matrix, **I** is an identity matrix, and σ_u_^2^ and σ_e_^2^ are the marked additive genetic and residual variances, respectively. A marker was declared significant if the *t*-test for its allele substitution effect presented *p* < 9.63 × 10^-8^ (Bonferroni correction).

### Whole Genome Sequence of Limonero Cattle

Genome sequence for individuals of the Limonero breed (*n* = 9) animals were obtained using Illumina Short Read Sequence technology (Bentley et al., 2008). The set of individuals included seven slick, one normal, and one long hair phenotype. Barcoded sequencing libraries were prepared from hair samples using Illumina TruSeq library kits and run as 6-plex pools on an Illumina HiSeq 2000. Sequence data quality was assessed with FastQC, and was aligned to the UMD3.1 (Zimin et al., 2009) reference genome (Ensembl release 73) using BWA MEM v0.7.9 (Li and Durbin, 2009). SNP and INDEL calls were generated using samtools v0.2.0 (Li et al., 2009) and the functional annotation of variant calls was accomplished using SNPeff 3.6c (build 2014-05-20) (Cingolani et al., 2012). The whole-genome sequences of the Limonero cattle including the metadata on the coat phenotype were deposited at https://www.ncbi.nlm.nih.gov/bioproject/ under BioProject PRJNA422135. The SNP called using these sequences within the critical genomic interval are available as downloads from https://github.com/USDA-ARS-AGIL/bos-taurus-slick-coat-variations.

### Targeted SNP Genotyping and Association Test

The candidate SNP identified sequencing the Limonero cattle were genotyped and tested for association in a larger validation sample (**Tables 1, 3**). Eight hundred and eleven animals were genotyped using multiplexed primer extension assays detected by mass spectrometry (Braun et al., 1997). Comparisons of coat phenotypes and genotypes were performed using the likelihood ratio test with the Williams correction for continuity (Sokal and Rohlf, 1981).

**Table 1:** Discordance between *PRLR* 20:39136558 GC > G and slick coats in all animals in this study.

	*PRLR* genotype		
Phenotype	GC homo^1^	G hete^2^	G homo^1^
WT coat	186	3	0
Slick coat	119	147	36

*^*1*^homo = homozygote; ^*2*^hete = heterozygote.*

### Predicted 3-D Structure of the Wild Type and Mutant PRLR

The GHR and PRLR belong to the superfamily class I cytokine, bovine and human protein reference sequences for both genes were aligned using Clustal Omega (Sievers et al., 2011). Protein folding was performed using Phyre2 in intensive mode (Kelley et al., 2015). The long form of the PRLR was used initially and once a model was found for the entire length, the PDB file of the long form of PRLR was used as a template to thread the truncation mutations. This would allow a specific comparison of the mutated proteins to the wild type protein and prevent spurious major changes in 3-D conformation.

## Results

Linkage analyses had previously located the slick locus of Senepol cattle to Bta20 and *PRLR* was identified early as a potential candidate gene (Mariasegaram et al., 2007). Additional studies had confirmed the location to the chromosomal region (Huson et al., 2014; Porto-Neto et al., 2014). A 1-bp deletion in the coding sequence of *PRLR* [20:39136558 GC > G] was first reported by Littlejohn et al. (2014), and this frameshift mutation was predicted to result in a truncated PRLR protein due to the gain of a stop codon just distal to the mutation. In our sample of Senepol and Senepol-admixed cattle, this 1-bp deletion showed concordance to slick phenotype.

However, genotyping of a range of breeds beyond Senepol that included South American criollo cattle breeds such as Limonero and Carora revealed that slick hair was not in perfect concordance with genotypes at the 20:39136558 GC > G deletion (**Table 1**). This validation panel of DNA samples included animals that were not expected to have the mutation, such as Holstein and Jersey. 119 of the genotyped cattle (*n* = 591) with slick coat phenotypes were discordant with the previously reported frameshift mutation. Most of the discordant animals were either Carora, Limonero or crossbred Romosinuano. These animals were re-phenotyped and re-genotyped to confirm their statuses.

**FIGURE 1:**
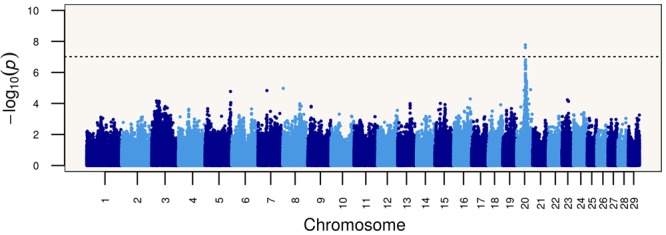
Genome-wide association study (GWAS) showing a strong signal of association between single nucleotide polymorphisms on bovine chromosome 20 at the *PRLR* gene and the slick phenotype in Limonero cattle.

Based on these results, we performed a genome-wide association study (GWAS) of the Limonero animals using Illumina BovineHD genotypes from 20 slick and 53 non-slick individuals. This analysis identified significant associations to markers in and around *PRLR* (**Figure 1**). Two SNP markers intronic to *PRLR* presented genome-wide significance, namely rs42551770 (20:39104658, *p* = 2.51 × 10^-8^) and rs137009256 (20:39110968, *p* = 1.67 × 10^-8^). These results suggest that the slick coat was likely due to a different mutation in the same gene previously identified (Littlejohn et al., 2014). Genome sequencing of Limonero animals (*n* = 9) identified three further nonsense variants leading to stop gained and a SNP which produced a synonymous mutation, all in the 11^th^ exon of *PRLR*. The latter SNP 20:39136518 had been described previously at the dbSNP, and has been also observed at the 1000 Bull Genomes project (run6) (Daetwyler et al., 2014). Among the three nonsense variants observed in the Limonero, only the SNP at 20:39136497 had been reported previously (**Table 2**). The new premature stop codon mutations were p.C440^∗^, p.S465^∗^, and p.R497^∗^. Interestingly, we confirmed the Senepol mutation in sequences from a crossbred Limonero. The previously described 1-bp frameshift deletion causes a p.A461L substitution, but it is the following codon that becomes the p.L462^∗^ premature stop codon.

**Table 2:** Observed variants on the bovine chromosome 20 at the *PRLR* gene.

Position	A1	A2	Consequence		Reference
39136497^a^	T	A	Stop gained, nonsense	p.C440^∗^	rs468544332, dbSNP (release 150)
39136518	C	A	Synonymous coding	p.A447	rs110971500, dbSNP (release 150)
39136558	GC	G	Frameshift	p.A461L	rs517047387, dbSNP (release 150)
39136571^b^	C	A	Stop gained, nonsense	p.S465^∗^	This article
39136666^b^	C	T	Stop gained, nonsense	p.R497^∗^	This article

*^*a*^SNP monomorphic in the validation sample. ^*b*^Polymorphic SNP in the validation samples, and used on further analyses.*

The new functional mutations in *PRLR* were tested across a set of 811 animals for concordance with the slick phenotype. The pC440^∗^ was monomorphic in the validation sample and was not included in further analyses. The p.R497^∗^ allele was also found in Carora animals. Neither of the three new nonsense mutations were found in Senepol cattle. Slick phenotypes of the previously discordant phenotype–genotype pairs could be correctly predicted 88.4 and 79.1% of the time in Limonero and Carora cattle, respectively, using Bayes theorem estimation (**Table 3**). We analyzed animals with slick coats that were wild type at the Senepol mutation and compared them to animals of the same breed that had wild type coats and were also wild type at the Senepol mutation. Only the Limonero and Carora breeds segregated both of the new truncating variants of *PRLR* and coat type (**Table 3**). In **Table 3**, animals were scored as heterozygous or homozygous for any of the truncation mutations, and where called heterozygous even if they were heterozygous for both truncation mutations.

**Table 3:** Association of new *PRLR* mutations to slick coats in the Limonero and Carora breeds in this study.

	*PRLR* genotype		
Phenotype	WT	Heterozygote^1^	Homozygote^2^
**Limonero**			
Slick coat	5	34	10
WT coat	15	5	1
**Carora**			
Slick coat	6	26	12
WT coat	3	5	5

*^*1*^Animal heterozygous for one or both of the new *PRLR* mutations. ^*2*^Animal homozygous for one of the new *PRLR* mutations. There were no animals homozygous for both mutations.*

Homozygotes for the new truncation mutations were identified and no animal had homozygous haplotype for both mutations. Of the 22 homozygotes for the new *PRLR* truncation mutations that were slick coated, 12 were Carora, and 10 were Limonero. However, there were 6 homozygotes that were not slick coated, 5 of which were Carora, and 1 was Limonero. The distribution of phenotypes and genotypes were divergent from the null (χ^2^ test *p*-value = 5.02 × 10^-6^, *n* = 127). Closer examination of the distribution of genotypes indicated a difference between the Limonero and Carora breeds. While the Limonero data by itself showed a strong divergence from the null (χ^2^ test *p*-value = 1.00 × 10^-6^, *n* = 70), the Carora data showed no divergence from the null expectation (χ^2^ test *p*-value = 0.24, *n* = 57), indicating that the divergence is driven by the data from the Limonero breed. Collapsing the table by genotype showed no divergence of breed by coat type, while collapsing the table by coat type showed no divergence between breed and genotype. Therefore, the difference in the effects seen between the two breeds is not due to differences in allele frequency or slick coat frequency between them.

**FIGURE 2:**
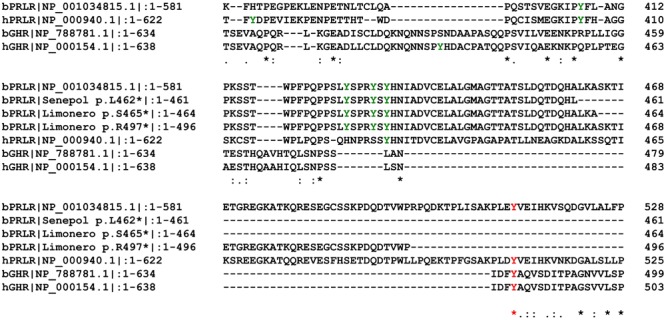
Clustal alignment of part of the cytoplasmic section of the bovine and human PRLR and GHR showing the location of truncated alleles highlighted in color with the tyrosine aa in green. Red after p.L462^∗^, brown after p.S465^∗^, and blue after p.R497^∗^.

**FIGURE 3:**
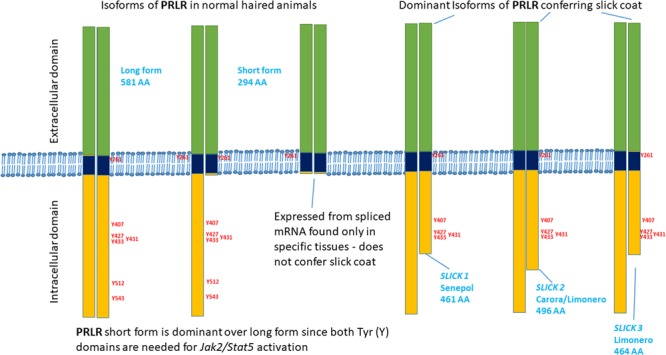
Schematic diagram of the truncated cytoplasmic domains of the PRLR mutations associated with slick coats.

All of the premature stop codons are expected to have identical or very similar functionality to the p.L462^∗^ mutation (**Figures 2, 3**). The location and relative length of each mutated sequence is shown using different color codes, with the tyrosine residues highlighted. Alignment of the PRLR and GHR amino acid sequences of cattle and human shows that all the premature stop codons occur after a strictly conserved asparagine at N434 of bovine PRLR. This also means that all truncated sequences have five tyrosine residues after the transmembrane domain, which is the hydrophobic section from T235 to L258. All mutated segments truncated before the conserved tyrosine residue at Y512, which is conserved across human and cattle GHR and PRLR. All truncated cattle PRLR sequences lack the same 2 of 7 tyrosine residues.

**FIGURE 4:**
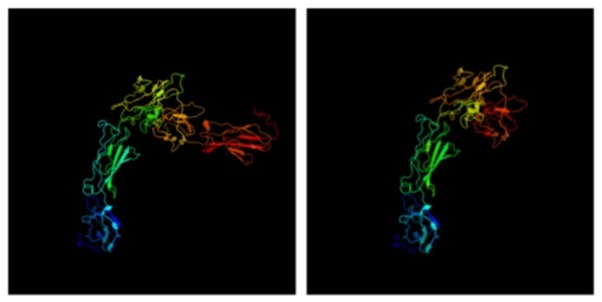
3-D structure of PRLR long form using Phyre2. The truncation mutations lack the final cluster of beta-strands shown in deep orange and red.

We modeled the 3-D protein structure of bovine PRLR using Phyre2 for both extracellular and cytoplasmic domains (**Figure 4**). In the protein databases, there were no long-form 3-D structures for either PRLR or GHR, only short forms. Most mappings of PRLR, therefore, show alignments to the short form. Nevertheless, there were reasonable matches to the cytoplasmic domain and a Protein Data Bank (PDB) format file for the modeled long form. This model showed that the two known extracellular clusters of alpha-helices and beta-strands are linked to two cytoplasmic clusters via the transmembrane domain. Using this PDB format file as a template, we inter-threaded the mutated long form of PRLR associated with the slick phenotype. Comparison of the image shows that one entire cluster of beta-strands, shown in red and deep orange in **Figure 4**, is missing from the truncated proteins (slick version). In conjunction with the loss of two tyrosine aa through truncation, there is also a clear, large difference in 3-D protein structure. It is known that PRLR dimerizes when it binds PRL (Chang et al., 1998), but it is not known what effect the lack of a cluster of beta-strands would have on the structure of the dimer and subsequent downstream signaling.

## Discussion

We have identified further mutations in the portion of *PRLR* encoding the cytoplasmic domain of the protein, and these mutations account for most genotype–phenotype discrepancies for the slick coat type. Our initial work confirmed the 1-bp deletion in *PRLR* first reported by Littlejohn et al. (2014). Application of that test to the Carora and Limonero breeds immediately showed that the primary *PRLR* mutation was absent in those animals. Whole-genome sequencing of Limonero cattle from Venezuela identified further stop codons in the same region of the *PRLR* sequence encoding the cytoplasmic portion of the protein receptor. Genotyping of these alleles explained most of the previous genotype–phenotype discrepancies. However, there remained a few animals with slick coats that did not have any of the three truncation mutations. Some of this non-concordance could be the result of indicine admixture or other non-detected mutation. Furthermore, there were individuals in both the Carora and Limonero breeds that had a wild type coat phenotype but possessed a *PRLR* truncation allele. Three animals that possessed the Senepol truncation allele also showed wild type coats. Even though the slick coat can be clearly identified by a trained person, it is still at certain degree a subjective measurement, also the high genetic variability at the PRLR locus and non-concordance of genotypes to phenotypes of some animals might suggest a potential complex inheritance mode of the phenotype. Nevertheless, these results show that the slick coats of criollo cattle do not all arise from introgression of the Senepol allele, but represent new mutations indigenous to those cattle.

The functional importance of the cytoplasmic segment of PRLR is well-known, and these slick mutations generate truncated proteins that should have nearly identical effects on protein function. The GHR and PRLR belong to the class I cytokine superfamily, they are assumed to be consequence of multiple gene duplication followed by divergent evolution (Iso-Touru et al., 2009). The conserved features of the two protein sequences are important as GH can still activate PRLR (Boutin et al., 1989; Dorato et al., 1992; Somers et al., 1994). The conserved functional regions encoded by *PRLR* and *GHR* have been experimentally validated, including the use of truncation mutations (Chang et al., 1998; Gadd and Clevenger, 2006), which have been shown to substantially change signal transduction dynamics of the prolactin receptor. This has led to computational predictions of the binding kinetics for PRLR (Liu and Brooks, 2011; Pang and Zhou, 2012). When prolactin binds its receptor, the receptor dimerizes, and the signal is transduced via associated signaling proteins, such as Jak2, Fyn, Grb2/Sos1, Raf, Vav, and Stat5 (Chang et al., 1998; Endo et al., 2003; Pang and Zhou, 2012). PRLR lacks its own intrinsic tyrosine kinase catalytic domain, but the tyrosine aa in the cytoplasmic domain are critical to this function (Chang et al., 1998). In addition to the tyrosine aa, certain sections of the cytoplasmic domains of GHR and PRLR have long been known to have much higher levels of evolutionary sequence conservation (Boutin et al., 1989; Kelly et al., 1992; Chang et al., 1998). These include sections known as box 1, V-box, box 2, and X-box motifs (Chang et al., 1998). The truncation mutations all occur after the box 1 through X-box motifs, after a conserved asparagine at N434, and occur in a part of the PRLR cytoplasmic sequence that is not conserved with GHR. All truncations occur before a conserved segment starting at P509 which included a tyrosine Y512 that is conserved across GHR and PRLR in humans and cattle. For all known functionally important domains, the truncation mutations are identical even though they have different lengths, and each mutation has the same reduced number of encoded tyrosine aa.

Our identification of these truncation mutations is a clear case of convergent evolution and highlights the substantial degree of mutational tolerance in a critical cell surface receptor protein. PRLR is ubiquitously distributed and it has functions well-beyond the reproductive-mammary gland axis (Foitzik et al., 2009), including effects on the skin, thermoregulation (Mundel et al., 2007), water and electrolyte balance (Bole-Feysot et al., 1998), metabolism (Fleenor et al., 2006), and circadian cycle (Bole-Feysot et al., 1998; Lincoln et al., 2006). These mutations have been increased in abundance probably through a combination of natural selection, because of the advantage conferred on survival in hot tropical environments, as well as artificial selection by farmers. Additional studies using larger sample sizes are needed to determine if there are subtle differences among phenotypes induced by the mutations. The identification of the genetic basis for the slick phenotype in Carora and Limonero cattle provides a broader set of genetic variation that can be widely used to improve heat tolerance in the tropics. The use of these animals to introduce new slick alleles where needed can now be enhanced using DNA tests.

## Ethics Statement

The animals sampled specifically for this project had their processes evaluated and approved by the Animal Ethics Committee from the University of Florida, United States (IACUC – 201203578 and 201408607). The additional cattle included in the analyses were part of previously reported experiments that were referred in the text.

## Author Contributions

TS conceived and led most of the experiments, directed all other researchers, and drafted the manuscript. LP-N, WB, and AR did experimental design, data analyses, and drafted the manuscript. DB, YU, SS, JS, JC, and DN analyzed the data. AL-H, MP, EJ, PH, SD, EC, NA, and JG performed the experimental planning, sampling, and phenotyping. All authors read and approved the manuscript.

## Conflict of Interest Statement

TS and YU are Associate Editors, JC and JG are Review Editors at Frontiers in Genetics, Livestock Genomics. The other authors declare that the research was conducted in the absence of any commercial or financial relationships that could be construed as a potential conflict of interest. The reviewer H-JM and handling Editor declared their shared affiliation.
